# CENTRAL HEPATECTOMY FOR BILIARY CYSTADENOMA: PARENCHYMA-SPARING APPROACH FOR BENIGN LESIONS

**DOI:** 10.1590/0102-6720201600040021

**Published:** 2016

**Authors:** Raphael L. C. ARAUJO, Danielle CESCONETTO, Vagner Birk JEISMANN, Gilton Marques FONSECA, Fabricio Ferreira COELHO, Jaime Arthur Pirola KRUGER, Paulo HERMAN

**Affiliations:** 1 Liver Surgery Unit, Hospital das Clínicas, University of São Paulo Medical School, São Paulo, SP; Brazil; 2 Department of Upper GI and HPB Surgery, Barretos Cancer Hospital, Barretos, SP, Brazil

**Keywords:** Hepatectomy, Cystadenoma

## INTRODUCTION

Central hepatectomy (CH) is also known as mesohepatectomy and means hepatic resection of segments 4, 5, and 8^9^. Hepatic lesions located in these segments may require extensive resections, such as right, left, extended right or extended left hemi-hepatectomies especially due to their relationship to major vascular and biliary structures. CH represents a potential risk of intraoperative bleeding, biliary injury, and risk of positive margins, but also represent the appealing concept of parenchyma sparing, furthermore in benign lesions.

Is reported a case of a symptomatic patient with a large complex cystic tumor who underwent a CH without tumor violation and no major postoperative complication. 

## CASE REPORT

A 61-year old female patient with history of choluria, acholic stools, jaundice and pain in the right upper abdominal quadrant had undergone a cholecystectomy and hepatic cyst unroofing by laparotomy in another institution, 30 months ago. Due to the cholestatic symptoms recurrence, she was refered to our center. 

**FIGURE 1:**
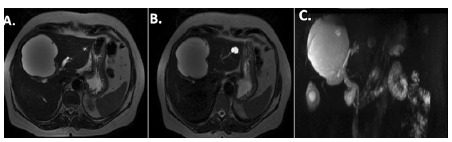
Pre-operative magnetic resonance image: A and B) axial image showing presence of central cystic lesion with thick wall and septum, in contact of hilar plate and placed in segments 4, 5, and 8; C) cholangio-resonance showing the contact to hilar plate and intra-hepatic dilation.

Abdominal MRI showed a cystic lesion in segment 4 with septa and thickened walls, and measuring 9.0 cm. The cyst was demonstrated as isosignal on T1 and hyperintense signal on T2. The confluence of left and right bile ducts was compressed by the cyst, which caused moderate bilateral dilation. The lateral limit of the cyst compressed the left hepatic artery and the left branch of the portal vein, while its lower limit compressed the right portal branch and the right hepatic artery. Other non-complex cystic lesions were scattered through the liver (Figure1). Laboratory tests showed increased canalicular enzymes and bilirubins and negative tumor markers. The case was reviewed at a weekly hepatobiliary multidisciplinary conference and the main hypothesis was a recurred biliary cystadenoma. In order to avoid a right trisectionectomy the decision was to perform a parenchymal preserving resection - central hepatectomy.

**FIGURE 2:**
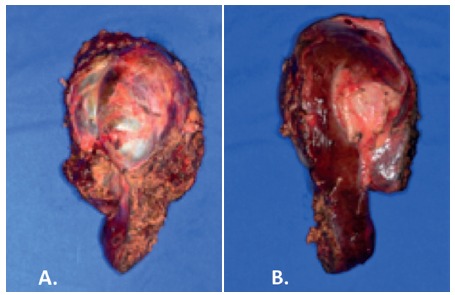
Operative image of central hepatectomy specimen: A) face in contact of hilar plate presenting sulcus of impressed by hilar plate; B) parietal face of liver

**FIGURE 3:**
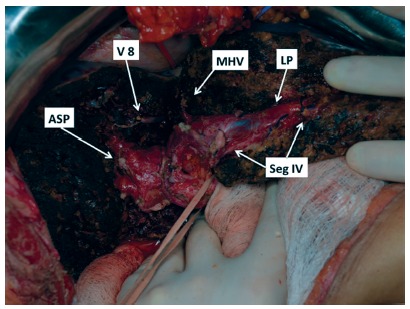
Sectional area of liver demonstrating hilar plate preserved by anatomical central hepatectomy (V 8=ligated vein from segment 8 into middle hepatic vein; right hepatic vein; MHV=middle hepatic vein; ASP=anterior sector pedicle; Seg IV=pedicles of segment 4A and 4B; LP=left pedicle)

**FIGURE 4:**
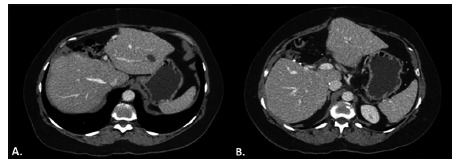
Eighteen months postoperative computerized tomography: A and B) showing compensatory hypertrophy without any recurrence or biliary dilation

During surgery, was confirmed the close relationship of the cyst and the hilar plate. Intraoperative ultrasound showed compression but not invasion of the hilar plate. The liver inflow was controlled with intrahepatic pedicle ligation of right anterior sector and segment 4. The cyst was separated from the hilar plate using ultrasonic dissector and bipolar eletrocoagulation, as demonstrated in Figures 2 and 3. Parenchyma transection was carried out with intermittent pedicle clamping (Pringle's maneuver). No blood transfusion was necessary. The postoperative course was only marked by a low volume biliary fistula conservatively managed with cavity drain placed during surgery (grade I - Dindo & Clavien classification)^2^. She was discharged on 8^th^ postoperative day. Pathological examination revealed a biliary cystadenoma presenting low-grade neoplasia with free margins. After 18 months of follow-up, the patient is doing well without either symptomatic or radiological recurrence (Figure 4). 

## DISCUSSION

CH is also known as mesohepatectomy, central hepatic resection, middle hepatectomy, middle hepatic lobectomy, and central bisectionectomy^9^. The putative risks of it compared to traditional major liver resections include a longer procedure time, greater intraoperative blood loss, higher risk of biliary and vascular complications, all mainly attributed to the proximity to hilar structures and the presence of two significant resection planes instead of a single one. Despite those concerns, this case highlighted that CH is safe and can be accomplished without significant morbidity. No significant differences for postoperative morbidity and mortality between CH and extend hepatectomy (EH) were demonstrated by Lee's systematic review^4^. Additionally, a recent case-matched study from the same author showed no differences in 90-day mortality, biliary leaks and postoperative liver failure^4^. Moreover, this study also showed longer length of stay, higher postoperative bilirubin and longer prothrombine time for patients who underwent extend hepatectomy. 

Liver parenchyma sparing aims to decrease the risk of postoperative liver failure, shorten recovering time, and allow re-hepatectomies in patients with high risk of recurrence. In patients with multifocal benign (adenomatosis) or malignant (colorectal liver metastases) diseases, when negative margins are sufficient, parenchyma sparing should be encouraged^1^.

Theoretically, the larger extension of liver transection plane in CH would promote longer procedures, and increasing risk of bleeding and biliary leaks. However, those concerns were not corroborated in comparative series of CH versus extend hepatectomy^4^. Moreover, CH presented shorter operative time (268 versus 299 min), and lower blood loss (882 vs. 1352 ml) when compared to extend hepatectomy. Regarding intraoperative bleeding, another useful tools applied to this case were the use of Pringle's maneuver and low central venous pressure^6^ ^,^ ^8^. The liver inflow control from Pringle's maneuver seems safe and avoids peri-operative blood transfusions, without negative impact in oncologic outcomes^10^. 

The conventional strategy to avoid postoperative liver failure involves determination of the future remnant liver volume and, when indicated, selective portal vein embolization together with degree of hypertrophy of remnant liver^5^. In cases of anticipated insuficient hypertrophy such as patients with severe steatosis, long-term chemotherapy and cirrhosis CH can be considered.

In this case, was faced a female with a symptomatic recurrent complex cystic lesion centrally located, a typical feature of biliary cystadenoma^8^. Surgery was indicated due to compression symptoms and the risk of malignancy. The option for a central hepatectomy represented a tailored procedure for this central located lesion.

In summary, this case highlights the importance of CH in the management of central liver lesions. CH is a technically demanding procedure, but its benefits overweight the fearsome complications of an extended resection and allow future re-interventions. 
